# Whole-genome sequencing of two chromogenic bacteria, *Exiguobacterium* spp. and *Pseudomonas chlororaphis*, isolated from environmental sources in Northern Colorado

**DOI:** 10.1128/mra.00091-26

**Published:** 2026-04-20

**Authors:** Taylor Melling, Carolina Mehaffy

**Affiliations:** 1 Department of Microbiology, Immunology and Pathology, Colorado State University 3447, Fort Collins, Colorado, USA; Indiana University, Bloomington, Bloomington, Indiana, USA

**Keywords:** pigment genes, antimicrobial resistance, whole genome

## Abstract

*Exiguobacterium* spp*.* (EA-TM-5) and *Pseudomonas chlororaphis* (PC-TM-6) were sequenced via Nanopore technology. De novo assembly resulted in one circular contig representing the complete genome of *P. chlororaphis* (PC-TM-6) and three circular contigs for *Exiguobacterium* spp*.* (EA-TM-5) representing its complete genome and two plasmids. Annotation revealed various antibiotic resistance genes.

## ANNOUNCEMENT

Soil and water samples were collected at Colorado coordinates 40°34′31.2″N 105°05′09.5″W and 40°26′30.4″N 104°48′21.1″W, respectively. Samples were inoculated into nutrient agar and incubated at room temperature (RT) for 48 hours. Selected colonies (EA-TM-5 from water and PC-TM-6 from soil) were streaked for isolation, and glycerol stocks from each isolate were made. EA-TM-5 produced an orange pigment, and PC-TM-6 produced a green pigment. 16S rRNA Sanger sequencing identified EA-TM-5 as *Exiguobacterium acetylicum*, a gram-positive, facultative anaerobe, rod-shaped bacterium (1). PC-TM-6 was identified as *Pseudomonas chlororaphis*, a gram-negative, aerobic, rod-shaped bacterium (1) via MALDI-ToF (Bruker) with a score of 2.34.

These isolates may have relevance in pigment production. *Exiguobacterium* species produce a carotenoid orange-yellow pigment that has the potential to have antimicrobial properties (2). *P. chlororaphis* is known to produce phenazines, which can be multiple colors depending on the specific compound and strain (3).

Isolates were plated on tryptic soy agar and incubated at RT for 72 hours and then grown in 5 mL of LB in a shaker at RT. Genomic DNA (gDNA) was isolated using the New England BioLabs Inc. Monarch Nucleic Acid Purification kit #T3010A, following the manufacturer’s instructions. gDNA was quantified by NanoDrop and Qubit and then sequenced at the Institute of Arctic Biology’s Genomics Core Lab using the Nanopore library kit SQK-RBK114-24 for 72 hours on a Gridion using flow cell FLO-MIN114. Reads were called with the Dorado v7.9.8 and the base calling algorithm dna_r10.4.1_e8.2_400bps_sup@v5.0.0.

Assembly and polishing were performed in the Galaxy server v25.1.rc1 (4, 5) using default parameters. Nanoplot v1.46.1+galaxy0 was used to assess quality control of reads (6). Reads were pre-processed using Porechop v0.2.4+galaxy1 (7). Reads shorter than 1,000 bp were removed using Filtlong v 0.3.1+galaxy0 (8). De novo genome assembly used Flye version 2.9.6+galaxy0 with Nano-hq settings (9). Bandage Image v2022.09+galaxy4 was used to visualize assemblies (10), and Quast v5.3.0+galaxy1 was used to provide quality metrics for assemblies (11). De novo assembly of isolate EA-TM-5 resulted in a total length of 3,327,795 bp and 3 circular contigs (*N*50 = 3,119,822). De novo assembly of isolate PC-TM-6 resulted in one circular contig (*N*50 = 6,824,689) (Table 1). Circularity was determined by Flye.

**TABLE 1:** Genome assembly, antibiotic resistance genes, and pigment-related genes of strains EA-TM-5 and PC-TM-6

Species	Raw reads QC	Trimmed and filter- ed reads QC	Contig	Length (bp)^*a*^	Coverage	GC (%)	Number of annotated CDS	Antibiotic resistance genes	Pigment-related genes
*Exiguobacterium* spp. (EA-TM-5) Genome Accession number: JBSRPW01 Reads accession number: SRR36386096	Number of reads = 75,449 *N*50 = 5,307 bp Median Q score = 14.1.	Number of reads= 36,533 N50 = 6,318 bp Median Q score= 17.6	1	3,119,822	46×	45.82	1,883	vanY gene in vanA cluster and vanY gene in vanM cluster	Carotenoid
			2	10,622	51×		0	None	None
			3	197,351	44×		47	None	None
*P. chlororaphis* (PC-TM-6) Genome Accession number: JBSVDE01 Reads accession number: SRR36386172	Number of reads = 17,591 *N*50 =17,502 bp Median Q score =15.7.	Number of reads = 13,041 *N*50 = 17.782 bp Median Q score =18.1	1	6,824,689	19×	62.82	3,681	- adeF - FosA8 - ArnT *-Streptomyces rimosus* otr(A) *-Pseudomonas aeruginosa* soxR - vanH gene in vanA cluster - rsmA - VanG	- Pf-5 pyoverdine - endophenazine A/endophenazine B - APE Vf

^*a*^ All contigs were circular.

**Fig 1:**
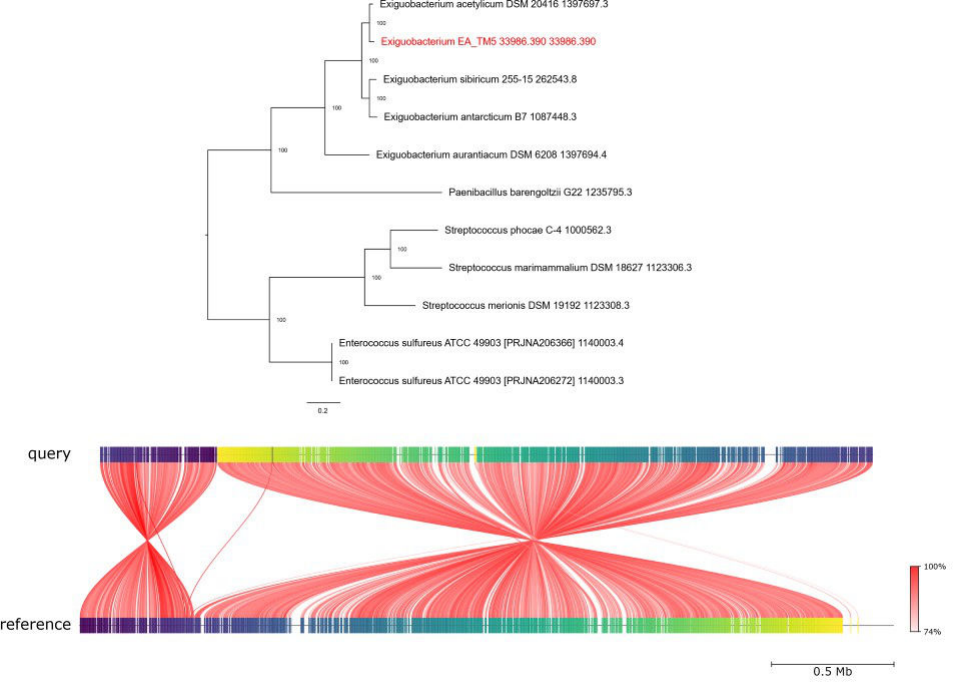
Top panel: Phylogenomic tree generated by the BV-BCR comprehensive analysis tool for the EA-TM-5, supporting the conclusion that EA-TM-5 may be a novel *Exiguobacterium* species closely related to *E. acetylicum*. Bottom panel: Results from FastANI (average nucleotide identity) between EA-TM-5 and *E. acetylicum* reference genome (accession: CP075897.1). FastANI indicated a 89.82%. Each red line segment denotes a reciprocal mapping between the “query” (Top, CP75897.1) and “reference” (EA-TM-5, bottom) genomes. Each red line segment denotes a reciprocal mapping between the query (top) and reference (bottom) genomes. The shade of red represents the ANI percentage. Colors on the query and reference genome (horizontal segments) represent orthologous fragments of 3,000 bp.

Visualization was performed in Proksee (12) using the assembled genomes’ fasta files. Genome annotation using the PGAP pipeline is available on NCBI. FastANI v1.34 (13) was run in Proksee using reference genomes CP075897.1 (*E. acetylicum*) and CP118151.1 (*P. chlororaphis*). FastANI resulted in an average nucleotide identity (ANI) for EA-TM-5 of 89.82% against *E. acetylicum* and 98.01% for PC-TM-6 against *P. chlororaphis*. Confirmation of species identification was performed in PubMLST (14). This analysis indicated EA-TM-5 to be *E. acetylicum (*81% support) and PC-TM-6 to be *P. chlororaphis (*100% support). The assembled genome of EA-TM-5 was further analyzed using the comprehensive analysis tool in BV-BCR (15), which indicated the closest related genome was *E. aceytilicum* via the core genome phylogenomic tree. Upon submission of the EA-TM-5 assembled genome to NCBI, the taxonomy was determined to be inconclusive. These results suggest that EAT-TM-5 may represent a novel species of *Exiguobacterium* and is not *E. acetylicum (*Fig. 1). Antibiotic resistance genes were identified using the CARD database (16) in Proksee, and AntiSMASH v8.0.4 was used to identify pigment-producing genes (17, 18) (Table 1).

## Data Availability

The Whole-Genome Shotgun project for *P. chlororaphis*, PC-TM-6 has been deposited in GenBank under the accession number PRJNA1377689, biosample SAMN53798171. The version described in this paper is the first version JBSVDE01. Reads can be accessed in Sequence Read Archive with accession number SRR36386172. The Whole-Genome Shotgun project for *Exyguobacterium* spp., EA-TM-5 has been deposited in GenBank under the accession number PRJNA1358277, biosample SAMN53107724. The version described in this paper is the first version JBSRPW01. Reads can be accessed in Sequence Read Archive with accession number SRR36386096.
